# Editorial

**Published:** 1899-02

**Authors:** 


### HOMŒOPATHIC PHYSICIAN,

A MONTHLY JOURNAL OF

HOMŒOPATHIC MATERIA MEDICA AND CLINICAL MEDICINE.

If our school ever gives up the strict inductive method of Hahnemann, we are lost, and deserve only to be mentioned as a caricature in the history of medicine."—CONSTANTINE HERING.

Vol. XIX. FEBRUARY, 1899. No. 2.

### EDITORIAL.

THE PRESIDENT OF THE INTERNATIONAL HAHNEMANNIAN ASSOCIATION desires to call the attention of the members to the announcement that the next meeting of the Association will be held at Niagara Falls instead of Cleveland as had been decided at the last meeting. The Secretary sent out a postal card circular announcing that there had been some objection raised to the meeting being held at Cleveland, and the members were desired to express their choice between Cleveland and Niagara Falls. The returns show an overwhelming preference for Niagara Falls, and therefore the meeting will be held at the Falls. The exact date and the hall of assembly will be announced later.

It is earnestly hoped that every member will make a supreme effort to be present so that a large meeting may be assured and an interesting interchange of knowledge and experience may be secured.

It is urgently desired that all members should feel it incumbent upon them to contribute papers recording their experience and thoughts, still further to promote the interest of the meeting.

When we reflect upon what Homœopathy is, and what its power and influence for good; when we reflect how far in advance of the science of the day it stands and what problems it freely and easily solves; what a boon it is to all who trust their health and their lives to its methods; what a solace to the poor and the aged, and what a safeguard it is to the physician who has to bear so much responsibility for those who commit themselves to his care; it would seem that it ought to fill every man's heart to overflowing with admiration and gratitude, and inspire his tongue to spontaneous outbursts of eloquence that would thrill his fellow-practitioners and stir them to reciprocal efforts.

"Out of the abundance of the heart the mouth speaketh." Therefore there should be no lack of essays, reports of cases, comments and reflections, that taken together would make the most interesting and instructive meeting ever held by the Association.

There is no doubt at all that we have the material among so many men all industriously and conscientiously practicing the principle. The only difficulty seems to be to get them quietly to recall the experience they have, meditate upon it until they are filled with its glories, then give vent to their feelings in some fervid address, some impassioned appeal to their fellow-members.

No two practitioners of the exact Hahnemannian method can casually meet without falling at once into an enthusiastic interchange of experiences upon the wondrous powers of their system, an interchange highly beneficial to both.

Now, why should not this enthusiasm swell in volume in the much wider field of a meeting of a large fellowship of such practitioners, each one of whom must be brimming over with the remembrance of what he has witnessed in his daily life during the past year, and, indeed, many years? Why should it not take the form of written records that will perpetuate his thoughts and his deeds in the cause, and thus increase the energy and stimulate the courage of his fellows in their chosen pursuit in life?

We recommend these thoughts to the attentive consideration of every member of the International Hahnemannian Association, and also to those readers of this journal who are not members.

We say to them, send in your applications for membership; be one of us, and infuse your own eloquence, your own enthusiasm into the vortex of friendly discussion and argument at our annual meetings.

